# Retrospective Analysis of the Prevalence and Risk Factors of Retinopathy of Prematurity in a Single-Center Cohort in Romania and Comparison with National and European Data

**DOI:** 10.3390/medicina61010149

**Published:** 2025-01-17

**Authors:** Zsuzsánna Simon-Szabó, Sándor Pál, Aliz Pándi, Zsuzsanna Gáll, Hajnal Finta, Zsuzsánna Incze-Bartha, Cristina Maki, Manuela Cucerea

**Affiliations:** 1 Department of Pathophysiology, Faculty of Medicine, George Emil Palade University of Medicine, Pharmacy, Science and Technology of Targu Mures, 540142 Târgu Mures, Romania; zsuzsanna.simon-szabo@umfst.ro; 2 Department of Transfusion Medicine, Department of Laboratory Medicine, Medical School, University of Pécs, 7624 Pécs, Hungary; 3 Faculty of General Medicine, George Emil Palade University of Medicine, Pharmacy, Science and Technology of Targu Mures, 540142 Târgu Mures, Romania; pandi.aliz.20@stud.umfst.ro; 4 Department of Neonatology, George Emil Palade University of Medicine, Pharmacy, Science, and Technology of Targu Mures, 540142 Târgu Mures, Romania; zsuzsanna.gall@umfst.ro (Z.G.); manuela.cucerea@umfst.ro (M.C.); 5 Department of Public Health and Health Management, George Emil Palade University of Medicine, Pharmacy Science and Technology of Targu-Mures, 540142 Târgu Mures, Romania; hajnal.finta@umfst.ro; 6 Department of Anatomy, George Emil Palade University of Medicine, Pharmacy, Science and Technology of Targu Mures, 540142 Târgu Mures, Romania; zsuzsanna.incze-barta@umfst.ro; 7 Department of Ophthalmology, Mures County Clinical Hospital, 540058 Târgu Mures, Romania; inapan1971@gmail.com

**Keywords:** retinopathy of prematurity (ROP), screening, incidence, risk factors, preterm infants

## Abstract

*Background and Objectives:* This study investigated and compared with European literature data the incidence, severity, and perinatal risk factors of retinopathy of prematurity (ROP) in preterm infants admitted to the Premature Department of Mureş County Clinical Hospital over a two-year period (January 2022–December 2023). *Materials and Methods*: ROP screening was performed in 96 infants (76.8%) according to professional guidelines. A literature review was conducted to compare our findings with national and European data. Significant differences were identified in comparisons with studies from Cluj-Napoca (*p* = 0.0125), Timișoara, and Bucharest (*p* < 0.0074), as well as Serbia and Croatia when stratified by gestational age limits. The variations in GA thresholds (32 vs. 34 weeks) between studies required stratified analyses to ensure meaningful comparisons. The included European studies provided data on screening criteria, prevalence, and associated risk factors, offering a comprehensive perspective on screening effectiveness. *Results:* Among the 149 admitted patients, 125 were preterm (n = 125). Of the screened patients, 20 (20.83%) infants were diagnosed with ROP, including 13 boys (65%) and 7 girls (35%), all requiring prolonged respiratory support, and 55% of them needed blood transfusion. The average birth weight of affected infants was 1030.5 g (550–1700 g ), and the mean gestational age was 28.3 weeks (25–34 weeks). In those found to have stage 2 and 3 ROP in zone II with plus disease (n = 6), intravitreal anti-VEGF injections and/or retinal laser treatments were performed. Notably, no cases of ROP-related blindness were recorded. *Conclusions:* To our knowledge, this is the first study to compare ROP prevalence and screening outcomes across Romanian national centers. Identified risk factors in this cohort, such as respiratory distress syndrome, oxygen therapy, blood transfusion, and intraventricular hemorrhage, are consistent with the existing literature data. These findings underscore the importance of standardized screening criteria and effective management protocols to prevent ROP-related blindness. The comparative approach of this study highlights the necessity of harmonized internationally applied criteria to facilitate robust comparisons and, more importantly, improve patient care outcomes.

## 1. Introduction

Retinopathy of prematurity (ROP) consists of abnormal vascularization of the retina in preterm infants, which can be interpreted as an arrest of normal neuronal and vascular development of the retina and is the result of abnormal compensatory mechanisms represented by abnormal vascular proliferation, vitreous hemorrhages, and, in the most severe form, tractional retinal detachment [1]. ROP is one of the leading causes of childhood blindness and visual impairment worldwide [2]. Given its multifactorial etiology, several risk factors have been identified over the last 7 decades, of which the two main risk factors are low birth weight and low gestational age. Several studies confirm that prenatal and perinatal conditions; genetic, maternal, and fetal factors; and postnatal comorbidities, as well as patient morbidity outcomes, all have an important role in the pathogenesis of ROP [3]. Over the past decades, neonatal care has undergone extraordinary development, thanks to numerous research efforts. One of the ROP-related findings was the use of high oxygen concentrations in closed incubators, which has resulted in an explosion in the number of ROP cases. There is no consensus on the optimal oxygen concentration, as higher oxygen concentrations increase the chances of survival and the incidence of ROP [1,4,5]. Since modern care is available in neonatal intensive care units and oxygen therapy is administered more carefully, retinopathy of prematurity occurs in most cases in very low gestational age (less than 28 weeks gestational age at birth) and very low birth weight neonates [1].

**Figure 1:**
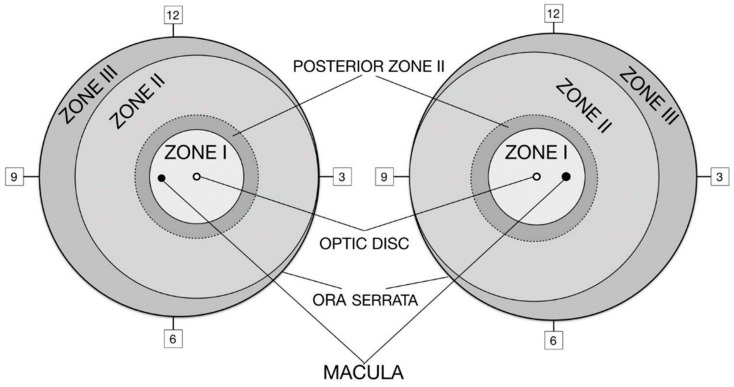
The zone borders and clock hour sectors are used to describe the location of vascularization and extent of retinopathy.

Based on the International Classification of Retinopathy of Prematurity, 3rd edition dating from 2019, ROP should be classified based on zone, plus disease, stage, and extent for each eye separately. “Zone” refers to the location of the disease, and “stage” is the description of the appearance of disease at the avascular–vascular junction, and separately describes the circumferential extent of disease. As well, “posterior zone II” and “notch” describe the incursion of disease into a more posterior zone, and there is also the sub-categorization of stage 5 as shown on Figure 1.

There are three retinal zones centered on the optic disc, according to which the most posterior retinal vascularization or ROP lesion will mark the zone of interest. Posterior zone II describes the region that begins at the margin of zone I–zone II and extends into zone II for two-disc diameters. “Notch” is used to describe an incursion by the ROP lesion of one–two clock hours into a more posterior zone. Plus disease refers to the appearance of dilation and tortuosity of retinal vessels, while pre-plus disease refers to abnormal vascular dilation and/or tortuosity that does not qualify yet as plus disease. Stages 1–3 define the acute disease, stage 4 is characterized by retinal detachment (4A—fovea attached; 4B—fovea detached), and stage 5 is total retinal detachment. Extent is described as the 12 sectors’ clockwise designation. The term “regression” is used to describe the course of ROP after treatment or spontaneous regression, which can be complete or incomplete regression, and “reactivation” is used to describe new ROP lesions or reactivation after laser-therapy or anti-vascular endothelial growth factor (anti-VEGF) treatment [6].

Timely screening and early diagnosis are both essential for the prevention of ROP-related blindness. In Romania, according to the national guideline, all premature babies born before or at 34 weeks of gestation and/or below 2000 g of birth weight, as well as those with perinatal risk factors predisposing them to the development of ROP, should be screened [7].

The primary objective of this study was to assess the prevalence and severity of ROP among preterm infants treated at the Preterm Unit of the Mureș County Clinical Hospital over 2 years. This cohort includes cases referred by other tertiary- or secondary-level hospitals. The secondary objective was to compare the prevalence of ROP observed in our cohort with data from similar studies conducted in Romania and other European countries or centers and to identify perinatal risk factors that potentially increased the risk of developing ROP in the study population.

## 2. Materials and Methods

Our retrospective study analyzed data collected from January 2022 to December 2023. During this period, 149 cases were admitted, of which 24 were term newborns and 29 preterm infants who did not undergo ROP screening, and consequently, these cases were excluded. To determine the prevalence of ROP in our clinic, we analyzed the data of 96 preterm infants with gestational ages less than or equal to 34 weeks and birth weight less than 2000 g who underwent ROP screening.

Screening was performed at 31 postmenstrual weeks for children born before 27 weeks of gestation and at 4–5 weeks after birth for children born at later gestational ages, whichever was later. Tropicamide and phenylephrine were used to dilate the pupils. A binocular indirect ophthalmoscope and speculum were used to examine the three zones of the retina to determine the stage (1–5), extent (based on the zones), and presence of any plus disease of the ROP. Careful monitoring was carried out every 1-2-3 weeks when needed.

### Literature Query

**Figure 2:**
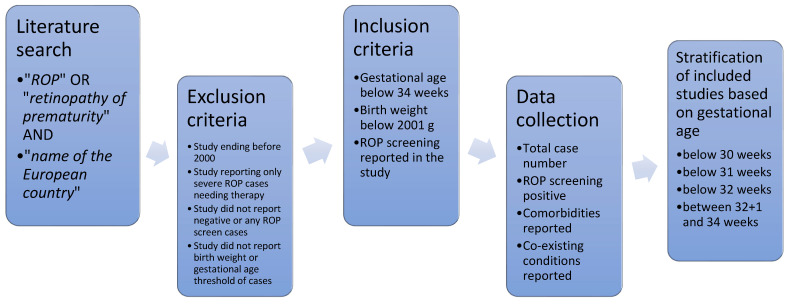
Flowchart diagram of study selection process.

A comprehensive literature search was conducted to compare our findings with previously reported data from Romania and Europe (Figure 2). For Romanian data, we used the search term “ROP incidence and Romania”, also in Romanian (“Retinopatia de prematuritate” and “Romania”). For European data, the search terms included “ROP incidence and Europe”, “ROP incidence”, and “retinopathy of prematurity and Europe”. In addition, search terms for specific European countries were applied, such as “retinopathy of prematurity and Serbia”, “retinopathy of prematurity and Norway”, and similar terms for other countries. The inclusion criteria for the comparative analysis were defined as a maximum birth weight of 2000 g, gestational age below 36 weeks, and a study period ending after the year 2000. Furthermore, studies reporting data on ROP screening, including case numbers screened for ROP, were also considered to be included in this analysis. The exclusion criteria were defined as studies that did not report the number of cases screened as negative for ROP or those that provided detailed data exclusively on treated ROP cases. The data regarding case numbers, ROP incidence, and case selection thresholds are presented in Table 1.

The study from Serbia included all preterm infants with a gestational age below 36 weeks or a birth weight below 2000 g. The studies were classified by the upper threshold of gestational age as “33 weeks or above” or below 32, 31, or 30 weeks.

Among the included European studies, articles from Belgium, Italy, Hungary, Norway, Turkey (2), and Serbia also reported the risk factors and/or comorbidities associated with ROP in the screened newborns.

In order to compare the reported incidences by the reviewed studies, the inclusion criteria for each study cohort, namely, gestational age, were taken into account. Comparisons were only performed if the inclusion criteria were identical, addressing potential differences arising from an unequal comparison.

The statistical analyses were performed using RStudio (v2024.04.2 + 764) with R (R Core Team 2024, Vienna, Austria) version 4.4.0 [38]. The data were represented either as categorical or as proportions. The confidence intervals of the reported incidences were calculated according to the Agresti–Coull method using the “binom” [39] library. The comparison with an inferential statistical method of the incidences of ROP were performed using the test of proportions with chi-squared distribution, but the research team relied only on the visual comparison by the graphical representation of incidences with confidence intervals. This approach has contributed to the high variability in the studies’ inclusion criteria, especially concerning the birth weight of the infants. Thus, stratification was performed using the reported gestational age thresholds. The graphical representation of the data was performed using the “ggplot2” [40] library. The threshold of significance was set to a *p* < 0.05 level. The confidence interval levels were set at 95%.

## 3. Results

A total of 60.41% of the study population was male. The average birth weight of the included cases was 1498.54 g (range: 550–2550 g), and the average gestational age at birth was 31.69 weeks (range: 25–36 weeks).

### 3.1. ROP Screening Results

A total of 20 (20.83%) preterm infants had any stage of ROP, including 13 boys (65%) and 7 girls (35%) with ROP at the following stages: 5 (25%) preterm infants had stage 1 ROP; 9 (45%) had stage 2 ROP; and 6 (30%) had stage 3 ROP. A total of 30% of the study cases were diagnosed with plus disease. The average birth weight in the ROP group was 1030.5 g (ranging between 550 and 1700 g), and the average gestational age at birth was 28.3 weeks (ranging between 25 and 34 weeks).

A total of 14 premature infants were diagnosed with type 2 ROP, including stage 1 and stage 2 ROP in zone II, without plus disease. These cases required only observation and follow-up. Six cases were found to have type 1 ROP, including stage 3 ROP in zone I and zone II with plus disease requiring immediate treatment within 72 h.

Laser treatment combined with intravitreal anti-VEGF injection (Bevacizumab, Avastin, Roche Pharma AG, Grenzach-Wyhlen, Germany) was applied to three infants, 3.13% of all screened premature babies, and 15% of those with ROP, respectively. Two children received Avastin injection, and one child was treated with laser photocoagulation. ROP regressed in all cases, and no stage 4 or 5 or retinal ablation occurred.

### 3.2. The Incidence of ROP

The studies included in the analysis for Romanian prevalence, along with the relevant data regarding case numbers and inclusion criteria, are summarized in Table 2. In Târgu Mureș, two previous studies were conducted in 2008–2012 referred to as Târgu Mureș (2) and 2008–2010 Târgu Mureș (3). These studies overlap to some extent in terms of the period covered, but the screening criteria for gestational age (GA) were different. We decided to include both studies to fully cover the screening criteria and keep all the results. No statistically significant differences were found between these two articles and our cohort (Târgu Mureș 1).

**Figure 3:**
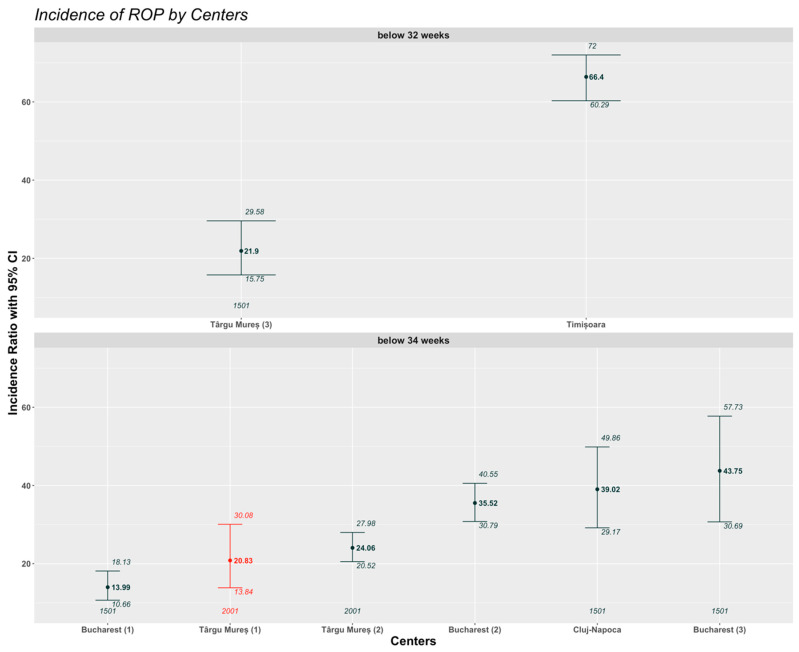
ROP incidence in Romanian centers grouped by GA criteria. Red color is showing our study results.

The prevalence of ROP in the Romanian studies based on GA as inclusion criteria is presented in Figure 3. Two of the studies have screened ROP below 32 weeks of GA; in the remaining five studies, the threshold for GA was 34 weeks. Due to the differences in the ROP screening criteria for GA, it was necessary to analyze and compare the same type of data separately. Therefore, the resulting studies were stratified by GA (Figure 3). Our study showed statistically significant differences compared to the study conducted in Cluj-Napoca (*p* = 0.0125), Timișoara, Bucharest (3) (*p* <0.0074338), and Bucharest (2) (*p* = 0.0089) (Table 2). We performed an extended search on the existing literature data regarding ROP prevalence in Europe. The studies included are listed in Table 3 with all the relevant information about the study period, total screened cases, identified ROP cases, inclusion criteria, and statistically significant differences compared to our findings (Table 3).

**Figure 4:**
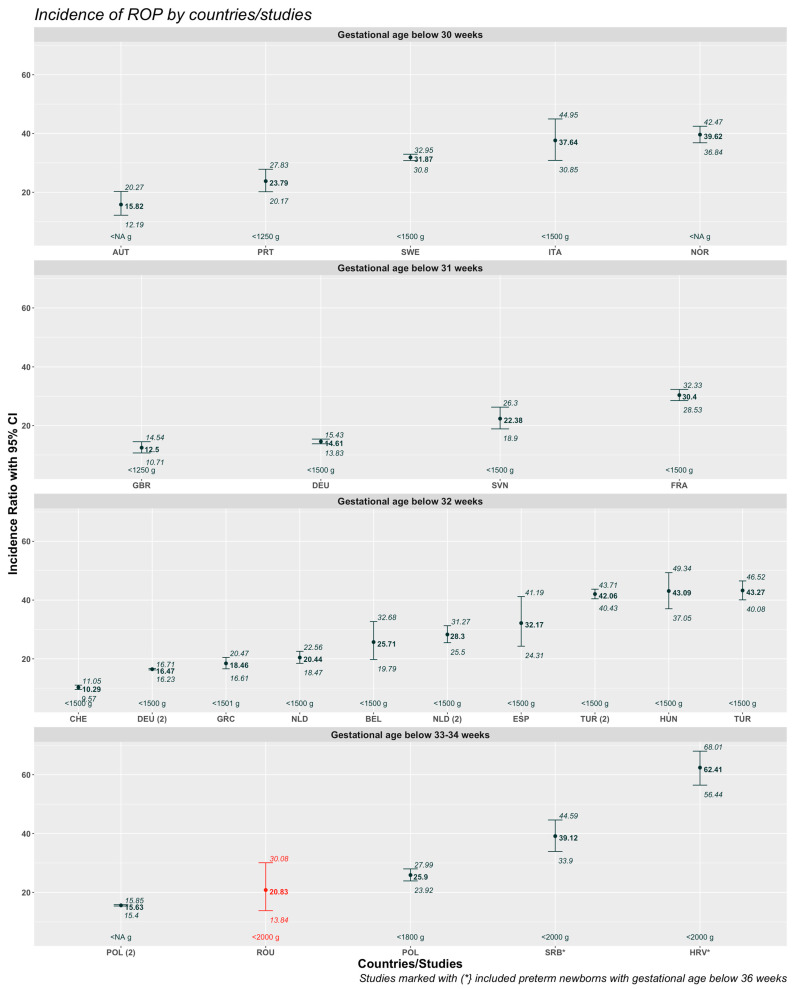
ROP incidence in European countries by GA criteria. Abbreviated country names. HRV: Croatia; SRB: Serbia; POL: Poland; ROU/TGM: Romania (Târgu Mureș); NOR: Norway; ITA: Italy; SWE: Sweden; AUT: Austria; PRT: Portugal; GBR: United Kingdom; FRA: France; DEU: Germany; SVN: Slovenia; TUR: Turkey; HUN: Hungary; CHE: Switzerland; ESP: Spain; NLD: Netherlands; BEL: Belgium; and GRC: Greece. Red is the result of our study.

Our cohort showed statistically significant differences compared to the studies in Serbia and Croatia when stratified according to similar screening criteria and adjusted for GA limits (Figure 4). Some of the studies reported information about risk factors and/or comorbidities associated with ROP in the screened newborns, as presented in Table 4.

The mean gestational age in our cohort was 28.3 weeks (range: 25–34 weeks) in the ROP group, compared to 32.56 weeks (range: 27–36 weeks) in those without ROP. All 20 premature infants diagnosed with ROP required prolonged oxygen supplementation, mainly due to surfactant deficiency and respiratory distress syndrome (RDS). They also had a lower GA. Among the subjects requiring invasive ventilatory support, a more advanced stage of ROP developed with plus disease.

In the study group, 95% of the cases had severe anemia, of which 55% required blood transfusions and 45% were treated by pharmacotherapy. In 65% of the cases, there were one or more heart defects, like PDA, PFO, or ASD. Bronchopulmonary dysplasia (BPD) and RDS developed in nine cases in the ROP group. Sepsis occurred in one case and intraventricular hemorrhage in two cases. Prolonged jaundice was found in 30% of the cases.

Among the 20 ROP cases, 5 cases were from twin pregnancies. In one of the sets of twins, one fetus died in utero. An analysis of maternal data during pregnancy showed a lack of maternal or perinatal care in 35% of cases (n = 7). Maternal pathologies in the non-follow-up pregnancies included sepsis (n = 1), infection (n = 1), obesity and diabetes (n = 1), hypertension (n = 1), and home birth (n = 2). Data on maternal pathology were not available in one case. All the other pregnancies received perinatal care, and no other pathology was identified during the antenatal period.

## 4. Discussion

This single-center study aimed to investigate ROP prevalence and to compare the findings with the available data published previously from Romania and other European countries. ROP prevalence in our cohort was significantly different from that of national studies from Cluj, Timișoara, and Bucharest. This can be explained by the differences in the time or period of the study, number of cases/births/years, and case complexity. The observed decrease in ROP incidence could partially be explained by the continuously improving quality of perinatal and neonatal circumstances and better neonatal intensive care management due to the wide range of research findings published.

A significant perinatal risk factor that has a major influence on the development of ROP is oxygen administration following birth, which has been substantially restricted considering the new findings. At the same time, there has been an important new approach to reducing oxygen concentration, using a lower concentration, or “the minimum necessary”, to achieve newborn stabilization, rather than 100% oxygen. For this reason, the time interval of the study period is important, as these changes were implemented gradually, and their effects manifested progressively. Studies reported that the duration of mechanical ventilation has a greater contribution to the development of ROP than the total duration of oxygen supplementation or any other method of oxygen administration but is not associated with the progression of ROP [41]. In our study, the incidence of ROP was similar to several national studies, which were conducted after 2010. The study from Timișoara showed a statistically significant difference in the prevalence of ROP, but the inclusion criteria for the ROP screening were set below 32 weeks of gestation.

In addition to the administration of oxygen, gestational age and birth weight are also considered to be important factors in the development of ROP and the influence of its progression [42]. The literature data show that improvements in neonatal care have led to an increase in the survival of extremely preterm infants, which has subsequently led to an increase in the particularly vulnerable group at risk of ROP [1,30]. Our study reported significant differences in the prevalence of ROP compared with studies in which the screening cut-off was set at or below 32 weeks of GA, as a lower GA and lower BW were associated with a higher risk of severe ROP and other pathologies, such as sepsis, respiratory distress syndrome, bronchopulmonary dysplasia, anemia, necrotizing enterocolitis, and intraventricular hemorrhage [3].

The incidence of ROP showed important variability in the national and European literature that was reviewed. This may be attributed to the differences in screening protocols between countries as well as significant socioeconomic discrepancies between different European countries and significant disparities in healthcare services between regions within a country, particularly in the area of neonatal intensive care. According to the national data, the lowest incidence of ROP was registered in Bucharest (13.98%) [10] and the highest in Timișoara (66.39%) [8]. Based on regional data, an article published in 2019 reported the incidence of ROP in Romania at an estimated percent of 40–50%, with an approximately 9–16% necessity of treatment for ROP and at least 100 children with blindness or impaired vision due to missed screening in the period of 2002–2017 [43]. In the absence of a national registry regarding ROP prevalence, based on the studies published in the past two decades, we can expect that the incidence is around that estimation. Our findings support this, and we can confirm that our single-center data did not change significantly compared to the two previous studies conducted in our center, and the ROP prevalence is very similar to that found in Bucharest (Figure 2) [11,13,14].

Prolonged oxygen administration, low GA and BW, anemia, BPD, and RDS were the main risk factors identified in our cohort, findings that are consistent with the international literature. Among the studies from European countries, oxygen support and transfusion events were reported in Belgium, Italy, Hungary, and Turkey. Low and very low birth weight preterm newborns are prone to anemia, which may require red blood cell transfusion. Since the development of ROP has been associated with transfusion events, a correlation was examined. In our cohort, transfusion events occurred in 55% of the ROP cases. In a study performed on a cohort of 565 very low birth weight newborns by Uberos et al., receiving three or more transfusions was associated with a 2.77 times higher risk of developing ROP and a 3.95 times higher risk of severe ROP [44].

Several studies included in this review investigated perinatal risk factors and comorbidities associated with ROP and highlighted a significant correlation between prolonged oxygen therapy and the severity of ROP. Specifically, the duration of invasive oxygen support was notably longer in preterm infants who developed severe forms of ROP. This finding underscores the importance of closely monitoring and optimizing oxygen therapy in preterm infants, as prolonged exposure may exacerbate the risk of developing severe ROP. Further research is needed to explore targeted interventions that could minimize oxygen-related damage while ensuring adequate respiratory support in this vulnerable population.

Our cohort findings indicate that preterm infants diagnosed with ROP commonly had at least one comorbidity, such as respiratory distress syndrome (RDS), severe anemia, congenital heart defects, or bronchopulmonary dysplasia (BPD). Additionally, a few cases exhibited intraventricular hemorrhage, and one instance of sepsis was noted during neonatal intensive care. These results align with the existing literature, which also reports a high prevalence of comorbidities among ROP cases [16,21,22,23].

Oxygen therapy emerged as a significant risk factor in the development of ROP. Recent evidence-based protocols have included recommendations aimed to minimize oxygen concentration to the “minimum necessary” to stabilize newborns, instead of using higher concentrations or even 100% oxygen. Gradual changes in oxygen administration practices are thought to be a contributing factor to the observed decreasing incidence of ROP over time. Moreover, the duration of mechanical ventilation has also been emphasized as a great contributor to ROP development, highlighting the importance of further improvement in optimizing respiratory support practices. The Regional Operational Programme for Romania has achieved significant financial allocations and a 93.5% absorption rate in the 2007–2013 financial period. The program aimed to reduce regional disparities and improve health infrastructure, which could be correlated with the improvement in neonatal care after that period [45].

The study findings must be interpreted within a broader socioeconomic context. Romania is a high-income country [46]; still, the infant mortality rate was the second highest in the European Union in 2019, highlighting disparities in healthcare quality and access. Maternal health and socioeconomic conditions such as maternal age, twin births, preeclampsia, and peripartum infections significantly influence neonatal clinical outcomes, also emphasized by Shulman et al. in a study performed in the United States [47]. This study reported that for 35% of the infants with ROP, the perinatal care was not adequate. Targeted interventions addressing socioeconomic disparities in neonatal and perinatal care are needed for the improvement in clinical outcomes in neonates.

A significant contribution of this study to the literature is that previously no comparison of ROP prevalence has been made between different centers in Romania and no comparison with European data was carried out.

This study has some limitations, relying on previously documented data that may be inaccurate or incomplete due to variability in medical documentation practices. The relatively reduced sample size may hinder the generalizability of our findings, particularly in comparison with European studies that included large sample sizes in their analysis. Socioeconomic factors and the quality of neonatal care were just briefly considered in this study, which probably had an impact on the incidence of retinopathy of prematurity and its comorbidities. Another limitation is that severe ROP cases were not reported separately in some studies, due to lack of classification by severity category, or they only reported a total number of ROP cases, so the number of severe ROP that required treatment remains uncertain.

A limitation of this study is the potential bias introduced by the selection criteria of the studies included in our analysis. These studies varied in their objectives, as they did not solely focus on retinopathy of prematurity (ROP) but also addressed other prematurity-related comorbidities or risk factors.

## 5. Conclusions

The national register used in several countries highlights the importance of introducing this type of monitoring to improve the effectiveness of childhood blindness prevention. At the same time, the introduction of a single European protocol would eliminate the differences between countries due to the different inclusion criteria of ROP screening, but it would not solve the differences that emerge due to differences in the quality and performance levels of the healthcare system in each country. The difficulty of comparing studies conducted at different time intervals and applying multiple inclusion criteria, even within the same country, underlines the necessity of a multicentric, multinational Europe-wide study focusing on ROP screening protocol. Prospective studies and multicentric collaborations are essential for standardizing data collection. The outcome of such a pilot study could be useful for the development of a Europe-wide ROP screening protocol based on a prevention algorithm that includes risk indicators predicting the likelihood of developing ROP and could also be useful for predicting the progression of ROP once it has developed.

## Figures and Tables

**Table 1:** Criteria of case selection and number of included cases of the analyzed European and Romanian studies. Abbreviations: NA—unreported/missing/unknown data.

Place of Study	Study Period	Included Cases	ROP Cases	Gestational Age (Weeks)	Birth Weight (Grams)
**National studies**					
Timișoara [8]	2017–2019	247	164	<32	≤2560
Cluj-Napoca [9]	15 months	82	32	<34	≤1500
Bucharest (1) [10]	2014	336	47	<34	<2500
Bucharest (2) [11]	2015–2016	366	130	<34	NA
Bucharest (3) [12]	2009	48	21	<34	<1500
Târgu Mureș (2) [13]	2008–2012	503	121	<34	≤2000
Târgu Mureș (3) [14]	2008–2010	137	30	<32	≤1500
**European international studies**					
Austria [15]	1999–2001	316	50	<27	<1320
Belgium [16]	1999–2000	175	45	<32	≤1500
France [17]	2011–2018	2247	683	<31	≤1500
Germany_1 [18]	2019	91551	15078	<32	≤1500
Germany_2 [19]	2011–2018	7496	1095	<31	≤1500
Greece [20]	2004–2020	1560	288	<32	≤1500
Hungary [21]	2017	246	106	<32	≤1500
Italy [22]	2017–2020	178	67	<30	≤1500
Norway [23]	2009–2017	1156	458	<28	<1000
Poland_1 [24]	2016–2019	1772	459	<33	≤1800
Poland_2 [25]	2012–2021	97,214	15190	<36	≤1800
Portugal [26]	2012–2020	475	113	<30	≤1250
Spain [27]	2004	115	37	<32	≤1500
Sweden [28]	2008–2021	7249	2310	<30	≤1500
Switzerland [29]	2006–2015	6472	602	<32	≤1500
The Netherlands_1 [30]	2017	1492	305	<32	≤1500
The Netherlands_2 [31]	2017	933	264	<32	≤1500
Turkey_1 [32]	2015–2017	906	392	<32	≤1500
Turkey_2 [33]	2016–2017	3490	1468	<32	≤1500
United Kingdom [34]	1990–2011	1152	144	<31	≤1250
Slovenia [35]	2015–2019	487	109	<32	≤1500
Serbia [36]	2006–2008	317	124	<36	≤2000
Croatia [37]	2009–2010	266	166	<36	≤2000

**Table 2:** Comparison of ROP prevalence with other studies in Romania.

Study	Total Cases	Number of ROP Cases	Incidence Mean Value % [95% CI]	*p*-Value
Timișoara	247	164	66.39% [60.29–72.00]	**<0.0001**
Bucharest (3)	48	21	43.75% [30.69–57.73]	**0.0074**
Bucharest (2)	366	130	35.51% [30.79–40.55]	**0.0089**
Cluj-Napoca	82	32	39.02% [ 29.17–49.86]	**0.0125**
Bucharest (1)	336	47	13.98% [10.66–18.13]	0.1404
Târgu Mureș (2)	503	121	24.05% [20.52–27.98]	0.5818
Târgu Mureș (3)	137	30	21.89% [15.75–29.58]	0.9739

**Table 3:** ROP prevalence in European countries, compared with the results of the current study.

Place of the Study	Interval	Incidence Mean Value % [95% CI]	*p*-Value *
HRV	2009–2010	64.40% [56.44–68.01]	**<0.0001**
SRB	2006–2008	39.11% [33.90–44.59]	**0.0015**
POL (2)	2012–2021	15.62% [15.39–15.85]	0.2062
POL	2016–2019	25.90% [23.91–27.99]	0.3231
ROU/TGM (1)	2022–2023	20.83% [13.83–30.07]	1.0000
NOR	2009–2017	39.61% [36.83–42.46]	**0.0004**
ITA	2017–2020	37.64% [30.85–44.95]	**0.0066**
SWE	2008–2021	31.86% [30.80–32.94]	**0.0280**
AUT	1999–2001	15.82% [12.19–20.27]	0.3223
PRT	2012–2020	23.78% [20.17–27.82]	0.6222
ITA (2)	2017–2020	37.64% [30.85–44.95]	**0.0066**
GBR	1990–2011	12.50% [10.71–14.53]	**0.0304**
FRA	2011–2018	30.39% [28.52–32.33]	0.0589
DEU	2019	14.60% [13.82–15.42]	0.1170
SVN	2015–2019	22.38% [18.89–26.29]	0.8418
TUR	2015–2017	43.26% [40.07–46.51]	**<0.0000**
TUR (2)	2016–2017	42.06% [40.43–43.70]	**<0.0000**
HUN	2017	43.08% [37.05–49.33]	**0.0002**
CHE	2006–2015	10.29% [9.573–11.05]	**0.0014**
ESP	2004	32.17% [24.31–41.18]	0.0906
NLD (2)	2017	28.29% [25.49–31.27]	0.1505
DEU (2)	2011–2018	16.47% [16.23–16.71]	0.3103
BEL	1999–2000	25.71% [19.78–32.68]	0.4524
GRC	2004–2020	18.46% [16.61–20.46]	0.6566
NLD	2017	20.44% [18.47–22.56]	1.0000

* Statistically significant differences are bold. Abbreviated country names: HRV: Croatia; SRB: Serbia; POL: Poland; ROU/TGM: Romania (Târgu Mureș); NOR: Norway; ITA: Italy; SWE: Sweden; AUT: Austria; PRT: Portugal; GBR: United Kingdom; FRA: France; DEU: Germany; SVN: Slovenia; TUR: Turkey; HUN: Hungary; CHE: Switzerland; ESP: Spain; NLD: Netherlands; BEL: Belgium; and GRC: Greece.

**Table 4:** Co-existing conditions reported in diagnosed ROP cases or severe ROP cases.

Study/Country	Belgium	Italy	Hungary	Norway	Turkey_2
Perinatal Risk Factors and Comorbidities	Control vs. ROP	Controls vs. ROP	Severe ROP (n = 28)	Controls vs. Severe ROP (n = 152)	Controls vs. Severe ROP (n = 152)
Parenteral nutrition	38.7 vs. 56.7 days	NA	NA	NA	NA
Surfactant administration	74% vs. 77%	NA	NA	NA	47% vs. 86%
Oxygen support	60.5 vs. 89.5 days	16 vs. 27.5 h	100%	NA	10 (0–171) days vs. 65 (0–308)
Chronic lung disease	79% vs. 94%	NA	NA	NA	NA
Central nervous system abnormalities	33% vs. 44%	8% vs. 16%	75%	5.9% vs. 10.5%	3.6% vs. 17.7
Systemic steroid administration	56% vs. 80%	NA	NA	NA	NA
Vasopressors	65% vs. 71%	NA	NA	17.1% vs. 37.5%	NA
Renal insufficiency	15% vs. 46%	NA	NA	NA	NA
Transfusions (event)	6.4 vs. 10.8	1.6 vs. 4.4	93%	NA	Two or more: 26% vs. 83%
Bronchopulmonary dysplasia	NA	22% vs. 36%	79%	49.4% vs. 80.9	67% vs. 13%
Sepsis	NA	31% vs. 39%	79%	NA	33% vs. 74%
Persistent ductus arteriosus	NA	56% vs. 58%	57%	30.5% vs. 57.2%	36% vs. 56%
Necrotizing enterocolitis	NA	3% vs. 10%	18%	5.9 vs. 10.5%	7% vs. 21%

Abbreviations: NA—not applicable; ROP: retinopathy of prematurity.

## Data Availability

Due to privacy and/or ethical restrictions, the data are unavailable. The corresponding author may be contacted regarding the data.
